# The Healing Effect of Arnebia Euchroma in Second Degree Burn Wounds in Rat as an Animal Model

**Published:** 2012-02-01

**Authors:** S Ashkani-Esfahani, M H Imanieh, M Khoshneviszadeh, A Meshksar, A Noorafshan, B Geramizadeh, S Ebrahimi, F Handjani, N Tanideh

**Affiliations:** 1 Student Research Committee, Shiraz University of Medical Sciences, Shiraz, Iran; 2 Medicinal and Natural Products Chemistry Research Center, Shiraz University of Medical Sciences, Shiraz, Iran; 3 Histomorphometry and Stereology Research Center, Shiraz University of Medical Sciences, Shiraz, Iran; 4 Organ Transplant Research Center, Shiraz University of Medical Sciences, Shiraz, Iran; 5 Medical Ethics Committee, Shiraz University of Medical Sciences, Shiraz, Iran; 6 Department of Dermatology, Shiraz University of Medical Sciences, Shiraz, Iran; 7 Stem Cell and Transgenic Technology Research Center, Department of Pharmacology, Shiraz University of Medical Sciences, Shiraz, Iran

**Keywords:** Healing, Arnebia euchroma, Burn, Wound, Rat

## Abstract

**Background:**

Finding more efficient agents with fewer side effects for treatment of burns has always been a concern for researchers. Silver sulfadiazine (SSD), apparently due to its antimicrobial effect, is still one of the most common prescribed agents. Previous studies suggested that Arnebia euchroma (AE) has shown antimicrobial and anti-inflammatory activities. This study investigates the healing effect of AE extract in comparison with SSD in second degree burn wounds.

**Methods:**

Fort eight female Wistar rats (220±20 g) were divided into four groups. Standard second degree burn wounds were induced on the back of their necks. One group was treated with SSD; two groups were treated with AE cream at concentrations of 10% (AE10) and 20% (AE20) and the control group which received no treatment. The duration of treatment was 28 days.

**Results:**

This study revealed that AE and SSD noticeably improved re-epithelization, fibroblasts proliferation, and collagen bundle synthesis and had a noticeable anti-inflammatory effect compared with the control group.

**Conclusion:**

Results of the present study revealed that Arnebia euchroma herbal extract was an effective treatment for second degree burn wounds when compared with SSD.

## Introduction

One of the serious causes of morbidity and mortality all over the world is thermal burn injuries, which results in a large amount of expenditure and costs in health care.[1] In a study conducted in the West Azerbaijan Province of Iran, the incidence rate of burn hospitalization was 21.6 per 100.000 patients.[2] In another survey in south west of Iran, the overall incidence rates of hospitalization and death were 13.4 and 4.6 per 100 000 person-years.[3] Such reports showed noticeable incidence and mortality rates caused by burn injuries and their complications. Depending on the area, depth and site of the burn, the treatment is different.[4] There are many treatment choices for topical therapy of burn wounds such as silver sulfadiazine ointment (SSD), mafenide acetate and silver nitrate. For distinctive situations; bacitracin, neomycin, polymyxine B, mupirocin, silver-impregnated dressings or biologic membranes (such as Biobrane) are also used.[5] Conventional agents, particularly SSD ointments are still of the most common prescribed medicines for burn injuries.[6] On the other hand, there is insufficient evidence to show that silver-containing dressings inhibit wound infection and promote wound healing.[7]

The Arnebia genus from the family of Boraginaceae has various species growing in Asia and the drier regions of northern Africa.[8][9] Very prominent chemical components, namely naphthoquinones, are found in the outer layer of the roots of more than 150 species of Arnebia genus. Naphthoquinones fraction is composed of water-insoluble pigments such as shikonin and alkanin and isohexenylnaphthazarin esters derivatives which have widespread pharmacological properties including anti-inflammatory,[9] antimicrobial,[10] and anti-tumoral activity.[11]

The purpose of this study was to determine the healing effect of Arnebia euchroma on second degree burn wounds in comparison to silver sulfadiazine ointment using pathological and unbiased stereological methods.

## Materials and Methods

Arnebia euchroma was collected (June 2009) from Yasuj city at altitude of 720 meters. A voucher specimen of the plant (Jafari MD, 151) was deposited at the Herbarium of Research Center of Agriculture and Natural Sources, Yasuj University of Medical Sciences, Yasuj, Iran. Extract of leaves and root of the plant was prepared using a method introduced by Kaith et al.[9] To facilitate the application of the extract, a vehicle gel was produced using a reported method.[12] The concentrated plant extract was introduced to the gel in 10% and 20% v/v.

Forty eight female Wistar rats (220±20 g, nonfasted) were randomly divided into 4 groups. One group was treated with silver sulfadiazine ointment, and two groups treated with Arnebia euchroma extract at concentration of 10% (AE10) and 20% (AE20), and the control group (C) received no treatment. A 2×3 cm2 standard second degree burn wound was induced on the posterior surface of animal’s neck under general anesthesia by using a method employed by Kaufman T et al.[12] Treatments were carried out every 24 hrs for 28 days.

All animal experiments were approved by the Animal Ethics Committee of Shiraz University of Medical Sciences and care was in accordance with Ethics Committee of Shiraz University of Medical Sciences guidelines.

All the specimens were formalin-fixed and stained with hematoxylin-eosin as well as Masson-trichrome. Slides were inspected for re-epithelization, granulation tissue formation and inflammation by using a reported scoring method.[4]

To determine the rate of wound area reduction, digital photographs were captured from the wound surfaces every other day. A standard ruler was laid at the wound level to find the magnification on the computer monitor. The wound area was calculated by using a method introduced by Gundersen HJ et al.[13] A full thickness circle of 10 mm was removed from the skin of the wounds, and then embedded in a cylindrical paraffin block. Five µm and 15 µm sections were provided and stained with both Hedenhain's azan and hematoxylin and eosin (H and E) staining.[13] Microscopic analyses were done using a video-microscopy system made up of a microscope (E-200, Nikon™, Japan) linked to a video camera, and a flat monitor.

**Figure 1:**
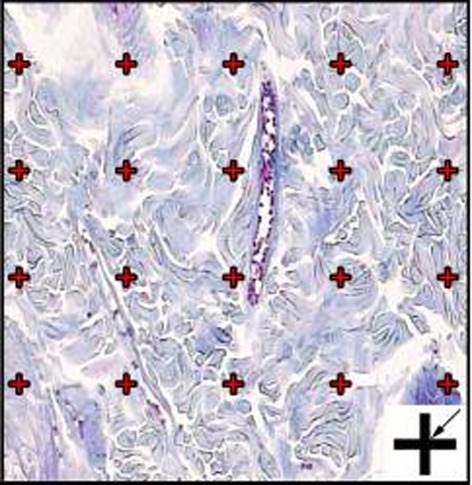
The volume density (Vv (collagen/dermis)) of the collagen fibers was estimated using a grid of points (designed at Histomorphometry and Stereology Research Centre, Shiraz University of Medical Sciences) on the live image of dermis. The total number of points hitting the bundles is counted and divided by the total number of points hitting the reference space (dermis). At the corner of this figure, a cross is presented. The right upper corner of the cross is considered as the point (arrow), and it is counted only if the right upper corner hits the tissue. (Hedenhain's azan stain) (×180)

**Figure 2:**
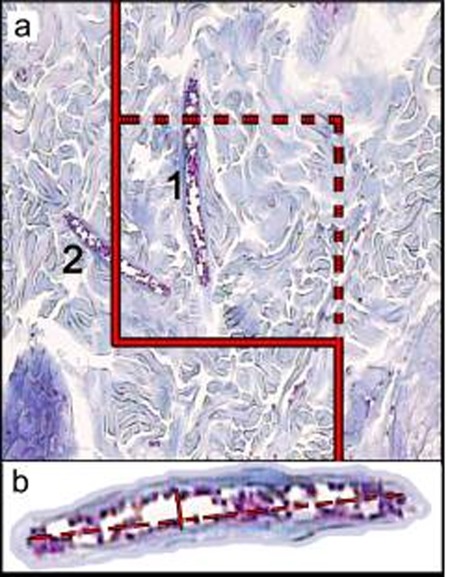
(a) An unbiased counting frame (designed at Histomorphometry and Stereology Research Centre, Shiraz University of Medical Sciences) was laid randomly on the monitor image of wound dermis for estimation of the length density (LV) and mean diameter of the vessels. Any vessel lied in the counting frame (1), or touched the inclusion borders (dotted lines) were selected. The vessels touched the exclusion borders (bold continuous lines), were ignored (2). (b) Mean diameter of the vessel were estimated by measuring the short axis of the vessel (short double arrow). (Hedenhain’s azan stain)

**Figure 3:**
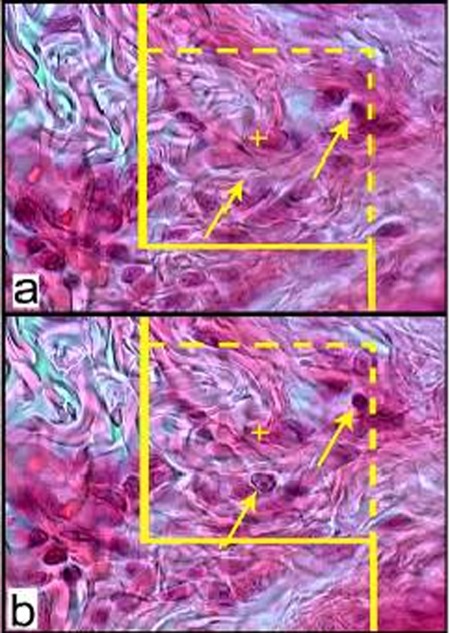
(a): An unbiased counting frame (designed at Histomorphometry and Stereology Research Centre, Shiraz University of Medical Sciences) superimposed on the monitor image of the sections to estimate the numerical density (NV) of the fibroblasts. The nucleuses are unclear at the first 5 µm optical section (height of disector) (b): As above, any nucleus lied in the counting frame or touched the inclusion borders (yellow dotted lines) and did not touch the exclusion borders (bold yellow lines) and come into maximal focus within the next traveling 5 µm optical section (height of disector) are counted (the two arrows). (Hedenhain’s azan stain ×2000)

The volume densities (Vv (structure/dermis)) of the collagen bundles were estimated at final magnification of 180 by using a reported method (Figure 1).[13] The length density of the vessels (Lv) and mean diameter of the vessels were estimated at final magnification of 180 by occupying a method introduced by Mühlfeld et al. (Figure 2).[14] For these purposes, 5 µm sections were employed. The numerical densities (NV; number of cell per unit volume) of fibroblasts were estimated by employing 15 µm sections at magnification of 450 on the monitor, and the “optical disector” method (Figure 3).[13][14]

Data were collected, analyzed and reported as mean and standard deviation (Mean±S.D.). Statistical comparisons between groups were carried out by using SPSS software (Version 16.0, Chicago, IL, USA). One-way ANOVA followed by Tukey’s post test =were used to analyze the data. P≤0.05 was considered as statistically significant.

## Results

Pathological analyses of specimens revealed that the rate of granulation tissue formation was not significantly different between groups which received treatments (p=0.91) but differred considerably in comparison to the control group (p=0.045). There was slight acute inflammation in group SSD, slight acute inflammation in AE10, moderate acute inflammation in AE20 and a mixture of intense acute and chronic inflammation in the control group. Re-epithelization had covered more than 50% of the wounds in AE10, AE20 and SSD groups; but in control group, the ratio was less than 50%.

According to stereological analyses results, AE20 showed slower wound contracture in comparison with AE10 and SSD groups. On the other hand, the average of reduction in wound areas by AE10 was 16.73 mm2 per day, by AE20 was 14.95 mm2 per day and by SSD group was 17.73 mm2 per day which are meaningful in comparison to the average of 5.01 mm2 per day in the control group (p<0.001). Fibroblast proliferation rates (Nv) were higher than C by SSD ~42% (p=0.032); by AE10 ~40% (p=0.031) and by AE20 ~55% (p=0.019). Fibroblast proliferation rate by AE20 was insignificantly higher than SSD and AE10 groups (p>0.8).

Total volume of collagen fibers in SSD, AE20 and AE10 groups were similar but were significantly different from C group (p=0.017;Table 1).

**Table 1:** Mean (SD) of the numerical density of the fibroblasts (×103 per mm^3^), volume densities of the collagen bundles (Vv; %) and vessels, length density (mm/mm^3^) and mean diameter (μm) of vessels in the dermis of the burned rats treated with 10% and 20% solution of arnebia euchroma (AE10 and AE20) , silver sulfadiazine (SSD) and control group.

**Groups **			**Parameters **		
	**Nucleus density (Fibroblasts; Nv)**	**Total collagen volume (Vv)**	**Total vessel volume**	**Length density of vessels (Lv)**	**Mean diameter of vessels**
Control	171.58 (59.23)^a^	0.82 (0.021)^a^	0.08 (0.019)	56.29 (9.65)	10.86 (2.02)
SSD	243.84 (39.32)	0.96 (0.035)	0.04 (0.034)	59.86 (15.44)	11.94 (2.79)
AE10	241.66 (43.57)	0.96 (0.028)	0.04 (0.026)	66.09 (22.54)	13.13 (3.58)
AE20	265.60 (58.12)	0.93 (0.028)	0.07 (0.027)	59.77 (13.87)	12.93 (2.98)

^a^ p<0.05, Control vs. SSD and AE10 and AE20

In comparison with the C group, length density of vessels (Lv) was ~7% higher by SSD, ~17% higher by AE10, and ~7% higher by AE20, which were not different significantly (p>0.1). Mean diameter of vessels in comparison to the control group was ~10% higher by SSD group (p=0.34), ~21% higher by AE10 (p=0.09) and ~19% higher by AE20 (p=0.11).

## Discussion

The antimicrobial activity of SSD is likely to be the main reason of its common administration.[6][15] Current reports suggest that silver-based products are better to be avoided due to their side effects and the investigators are making efforts to detect better topical antimicrobial products.[7] Recent studies revealed that SSD ointment has had positive effects on proliferation of fibroblasts which are the main source of collagen and fibronectin.[16] Also positive stimulatory efficacies of silver sulfadiazine on angiogenesis, epithelization and promotion of granulation tissue formation have previously been reported.[17][18][19][20][21] Pirbalouti and colleagues had investigated the healing effect of AE in burn wounds (not a specific type of wound) based on pathological analyses; they also reported the anti-inflammatory effect of AE as well as its significant impact on fibroblast proliferation and collagen synthesis.[22] In another study, Nikzad and colleagues reported the positive effect of AE on density of blood vessels based on morphometrical analysis, but results of their research did not show any significant healing effect of AE’s leaf in wound healing process.[23]

In this investigation, microscopic study of specimens showed higher rates of granulation tissue formation that improved in all treatment groups. Antiinflammatory response in AE10 and SSD groups were more efficient than AE20; however, AE20 had a better response to inflammation in comparison with the control group. Although wound closure rate and re-epithelization in AE20 were insignificantly weaker than AE10 and SSD, statistical analyses revealed that silver sulfadiazine and Arnebia euchroma had similar stimulatory impact on wound contracture. Both AE and SSD had significantly stimulatory influence on fibroblast proliferation, collagen bundle synthesis, and revascularization; however, Arnebia euchroma showed higher induction of neovascularization which is of considerable prominence.

Despite many researches on the wound healing effect of AE, there were no conducted studies based on both stereological and pathological analyses evaluating the impact of AE on second degree burn wounds. In addition, as mentioned above, there were some controversies among the results obtained by previous investigations. Results of this study showed that wound healing by Arnebia euchroma was relatively comparable to treatment with SSD; however, further clinical studies are required to prove the efficacy of this herbal medicine in human model.
